# Facial UV photo imaging for skin pigmentation assessment using conditional generative adversarial networks

**DOI:** 10.1038/s41598-020-79995-4

**Published:** 2021-01-13

**Authors:** Kaname Kojima, Kosuke Shido, Gen Tamiya, Kenshi Yamasaki, Kengo Kinoshita, Setsuya Aiba

**Affiliations:** 1 Tohoku Medical Megabank Organization, Tohoku University, Sendai, Miyagi Japan; 2 RIKEN Center for Advanced Intelligence Project, Chuo-ku, Tokyo Japan; 3 Department of Dermatology, Tohoku University Graduate School of Medicine, Sendai, Miyagi Japan; 4 Tohoku University Graduate School of Information Sciences, Sendai, Miyagi Japan; 5 Institute of Development, Aging and Cancer, Tohoku University, Sendai, Miyagi Japan; 6 Advanced Research Center for Innovations in Next-Generation Medicine, Tohoku University, Sendai, Miyagi Japan

**Keywords:** Medical imaging, Risk factors, Skin manifestations

## Abstract

Skin pigmentation is associated with skin damages and skin cancers, and ultraviolet (UV) photography is used as a minimally invasive mean for the assessment of pigmentation. Since UV photography equipment is not usually available in general practice, technologies emphasizing pigmentation in color photo images are desired for daily care. We propose a new method using conditional generative adversarial networks, named UV-photo Net, to generate synthetic UV images from color photo images. Evaluations using color and UV photo image pairs taken by a UV photography system demonstrated that pigment spots were well reproduced in synthetic UV images by UV-photo Net, and some of the reproduced pigment spots were difficult to be recognized in color photo images. In the pigment spot detection analysis, the rate of pigment spot areas in cheek regions for synthetic UV images was highly correlated with the rate for UV photo images (Pearson’s correlation coefficient 0.92). We also demonstrated that UV-photo Net was effective for floating up pigment spots for photo images taken by a smartphone camera. UV-photo Net enables an easy assessment of pigmentation from color photo images and will promote self-care of skin damages and early signs of skin cancers for preventive medicine.

## Introduction

Skin hyperpigmentary disorders such as pigment spots and freckles are caused by melanin increase in epidermis and dermis. Hyperpigmentary disorders are classified into congenital pigmentary disorders including café-au-lait spot and nevus spilus or acquired pigmentary disorders including ephelides, melasma, and senile lentigo. Accumulated skin damages by aging and ultraviolet (UV) radiation accelerate acquired skin pigmentation^1,2^, and such accumulated skin damages also cause skin carcinogenesis such as actinic keratosis, squamous cell carcinoma, and melanoma^3^. Early signs of skin cancers therefore can be detectible by estimating acquired skin pigmentation, which reflects accumulated UV skin damages. However, it is difficult to detect early signs of UV skin damages and pigmentary disorders by imaging techniques under visible light. UV light is known to emphasize pigment spots from the absorption of UV light in melanin pigment^4^, and UV photography, a photographic process using light from the UV spectrum only, is used as a minimally invasive and accurate mean for the detection of pigmentary disorders. However, since UV photography equipment is not usually placed in general practice, it is desired to develop technologies that detect pigment areas on conventional color photo images taken under visible light in order to promote the assessment of skin pigmentation and early signs of skin cancers in daily life.

The recent development of deep learning technologies enables highly accurate image analysis in various fields. Remarkable progresses have been achieved particularly in medical image analysis by the deep learning technologies such as lesion detection in x-ray images, histopathological image analysis, and disease name classification for clinical photo images^5–11^. The increase of both computational resources and manually curated annotation data enables deep learning-based methods to lead accurate diagnosis results. Indeed, some of deep learning-based methods competed with medical specialists and achieved more accurate diagnosis results under certain conditions. Among the deep learning technologies, generative adversarial network (GAN) is a widely used learning framework for machine learning models to generate synthetic images^12^, and there exist various applications using GAN for medical data analysis^13–16^. Under GAN, deep learning models are trained to generate synthetic images that are likely to exist as images in some specified domain. For example, if celebrity faces are specified as the domain, the trained deep neural network under GAN generates synthetic celebrity face images that are likely to exist in the real world^17^. As an extension of GAN, conditional GAN (CGAN) has been proposed to convert input images to images that belong to some specified domain^18^.

CGAN considers two types of image domains, input domain and target domain. The deep learning model trained under CGAN takes input images that belong to the input domain and generates synthetic images suitable for the target domain based on the input images. An application of CGAN is colorization of grayscale images by setting grayscale images and color images as the input and target domains, respectively. CGAN also can be used to generate sketch images from color photo images by setting color photo images and sketch images as the input and target domains, respectively. We consider that CGAN is a promising technology to assess skin pigmentation by generating synthetic UV skin images that float up UV-damaged areas from general photo images taken under visible light. We thus propose a new CGAN-based deep learning method, named UV-photo Net, to generate synthetic UV photo images from face skin photo images taken by conventional digital cameras.

In UV-photo Net, color and UV photo images are set as the input and target domains, respectively, and then the deep neural network trained under CGAN generates synthetic UV-photo images from conventional digital color photo images. The assessment of UV skin damages and pigmentary disorders is hence enabled from the synthetic UV-photo images without UV photography equipment. Although the awareness for the risk of sun exposure is low even in the high-risk group for sun exposure such as farmers^19^, UV-photo Net will contribute to the promotion of self-assessment of UV skin damages and susceptibility to sun exposure, which is important for the detection of early signs of skin cancers as well as the enlightenment for preventive medicine.

**Figure 1:**
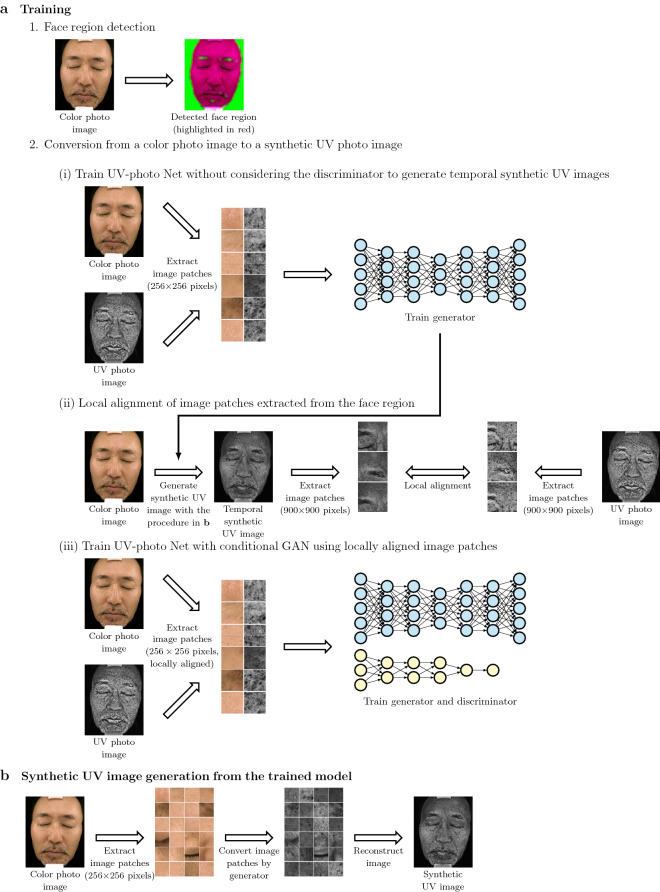
Outline of the training process of UV-photo Net. (**a**) The training process of UV-photo Net is comprised of (**a**-1) face region detection, (**a**-2-i) training for temporal synthetic UV image generation, (**a**-2-ii) local alignment of temporal synthetic UV images and UV photo images, and (**a**-2-iii) training under CGAN using locally aligned image patches. (**b**) Synthetic UV image generation from color photo images by assembling the image patches converted by UV-photo Net.

## Results and discussion

### Overview of training process of UV-photo Net

UV-photo Net converts color photo images to synthetic UV photo images using a deep neural network model called U-net, which is one of the widely used deep neural network structures for image conversion and image segmentation^20^. The size of color photo images considered in UV-photo Net is thousands of pixels in both width and height. Since whole color photo images are too large to handle directly in U-net, we used pairs of color and UV photo image patches of 256 ×256 pixels from face regions to train U-net. We trained U-net under CGAN by considering sets of color photo images and UV-photo images as the input and target domains, respectively. Training under CGAN considers two types of models called generator and discriminator. U-net serves as the generator in UV-photo Net to generate synthetic UV image patches from color photo image patches. The discriminator in UV-photo Net classifies the original UV photo image patches and synthetic UV image patches, and the loss based on the classification results from the discriminator is considered in the training process of the generator. With the loss from the discriminator, the generator is expected to re-generate synthetic UV image patches that are more perceptually realistic as UV photo image patches.

**Figure 2:**
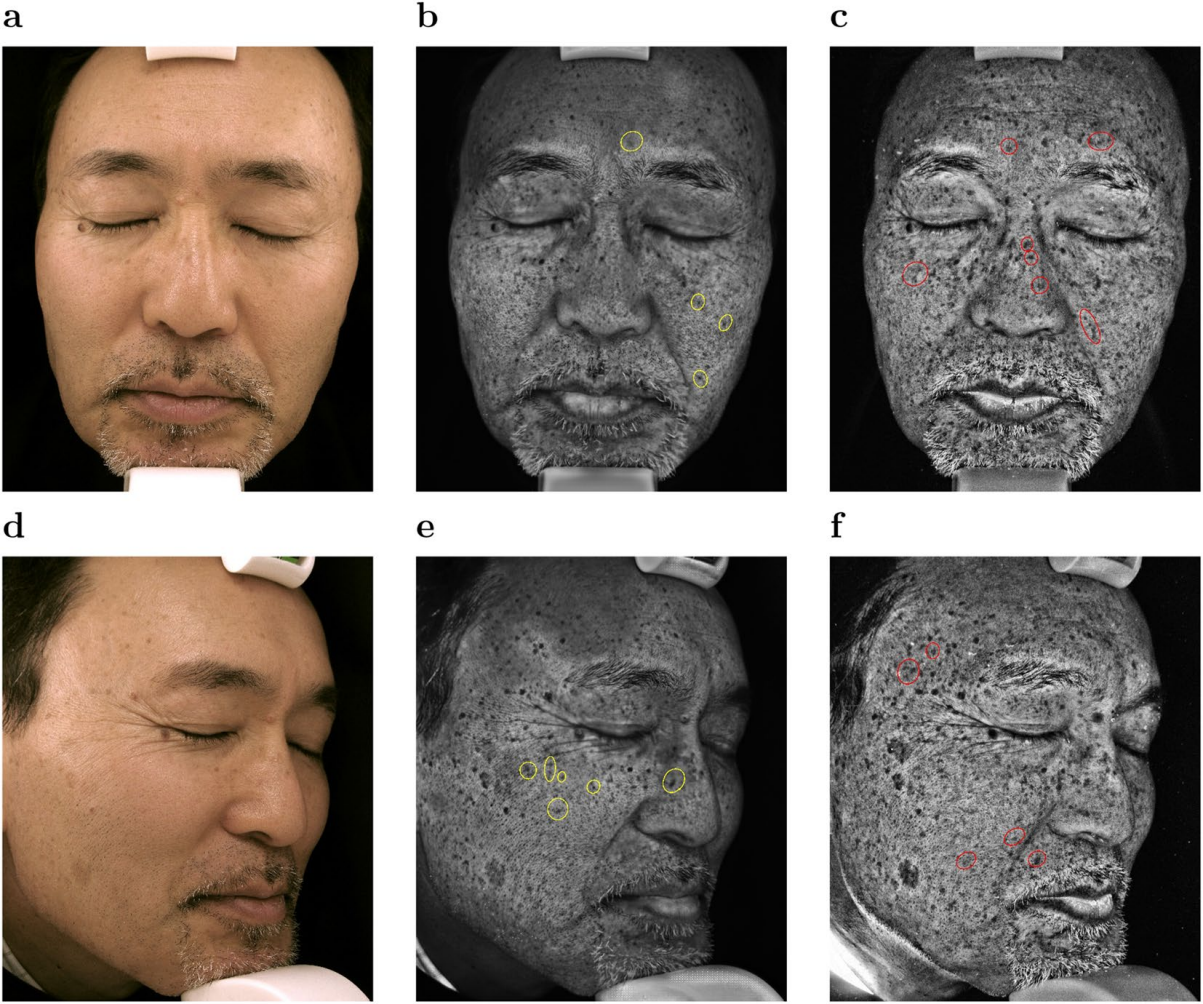
Color photo images of a front face and a right profile face (**a**,**d**), synthetic UV images generated by UV-photo Net (**b**,**e**), and UV photo images (**c**,**f**) for a subject.

Color photo images and their corresponding UV photo images are not matched completely in pixel-level due to the subtle movement of subjects in photographing time interval, and such pixel-level mismatches could cause blurred points in synthetic UV images. We thus considered the following steps to train UV-photo Net as shown in Fig. 1:

1. Detect face regions by a deep neural network, from which color and UV photo image patches are extracted for training data of UV-photo Net (Fig. 1a-1).
2. Train U-net without considering the loss from the discriminator to obtain temporal synthetic UV images from color photo images (Fig. 1a-2-i).
3. Align large image patches of 900 × 900 pixels from the temporal synthetic UV images and their corresponding UV photo images, and reflect the alignment results to their corresponding color photo images (Fig. 1a-2-ii).
4. Train U-net under CGAN using the locally aligned color and UV photo image patches (Fig. 1a-2-iii).

Since digital information of color and UV photo images is quite different, it is difficult to directly align color and UV photo image patches at pixel levels. We thus proposed to prepare temporal synthetic UV images from color photo images, which were expected to be the digital information comparable to UV photo images (Fig. 1a-2-i). We then used the temporal synthetic UV images for the alignment with UV photo images (Fig. 1a-2-ii). For the generation of the temporal synthetic UV images, we used U-net trained without considering the discriminator. The local alignment results for the temporal synthetic UV images were reflected to the corresponding color photo images. We then used the locally aligned color and UV photo image patches of $256\times 256$ pixels to train deep learning models for the generator and the discriminator (Fig. 1a-2-iii).

In the process of synthetic UV image generation, UV-photo Net first converted color photo image patches of $256\times 256$ pixels into synthetic UV image patches by the trained U-net (Fig. 1b). UV-photo Net then assembled the synthetic UV image patches to reconstruct whole synthetic UV images.

### Evaluation

We used color and UV photo image pairs of 184 Japanese individuals taken with VISIA Skin Analysis System (Canfield Imaging Systems, Fairfield, NJ, United States)^21^. We divided the 184 individuals into 160 individuals as training samples and the remaining 24 individuals as test samples. Age distributions of the training and test samples are shown in Table 1. Among the 160 training samples, we used color and UV photo image pairs taking front face appearances of 150 samples as a training dataset, and used image pairs for the remaining 10 samples as a validation dataset. The width and height of photo images taken with the VISIA system in our datasets is $3,456\times 5,184$ pixels, $3,000\times 4,000$ pixels, or $2,964\times 3,560$ pixels. We trained the deep neural network models by iteratively updating their parameters using the training dataset. The validation dataset was used for early stopping of the iteration.

**Table 1:** Age distributions of training and test samples.

Age group (years)	Training samples	Test samples
$10{-}19$	1	0
$20{-}29$	6	1
$30{-}39$	33	6
$40{-}49$	39	4
$50{-}59$	30	7
$60{-}69$	35	5
$70{-}79$	13	1
$80{-}89$	3	0

#### Evaluation in terms of per-pixel L1 loss and Fréchet Inception Distance

We used the color and UV photo image pairs of the 24 test samples to evaluate the accuracy of UV-photo Net. In order to examine the effectiveness of CGAN and the local alignment, we compared four training conditions; UV-photo Net, UV-photo Net trained without the discriminator, UV-photo Net trained without the local alignment, and UV-photo Net trained without both the discriminator and the local alignment. Table 2 shows per-pixel L1 loss and Fréchet Inception Distance (FID) of synthetic UV images generated in the four training conditions. We defined per-pixel L1 loss as the median of pixel-wise absolute distance between the synthetic UV images and the original UV photo images. Per-pixel L1 loss ranges from 0 to 255. Although the mean can be considered for the aggregation of the pixel-wise absolute distance instead of the median, we here adopted the median to avoid the influence of outlier values. We also summarized the per-pixel L1 losses obtained by the use of the mean in Supplementary Table 1. Note that the use of the mean or the median did not affect largely the superiority between the methods or the conditions.

FID represents the distance of two image distributions and is used for evaluating perceptual quality of synthetic UV images^22^. FID is obtained by calculating the distance of multivariate normal distributions of two types of images in the feature space of some deep neural network model with the following formula:

$$\begin{aligned} |\varvec{\mu }_1 - \varvec{\mu }_2|^2 + \text{ tr }\left( \Sigma _1 + \Sigma _2 - 2 (\Sigma _1 \Sigma _2)^{1/2}\right) , \end{aligned}$$

where $\varvec{\mu }_1$ and $\Sigma _1$ are respectively the mean and covariance matrix of the features for one type of images, and $\varvec{\mu }_2$ and $\Sigma _2$ are respectively the mean and covariance matrix of the features for the other type of images. In accordance with the FID calculation in^22^, we applied image patches of 256 × 256 pixels to Inception-v3^23^ trained on the ILSVRC-2012-CLS image classification dataset (http://www.image-net.org/challenges/LSVRC/2012/) and obtained the mean and covariance in the final layer of Inception-v3 to calculate FID values.

Both per-pixel L1 loss and FID were calculated in face regions, and the smaller value is better for both evaluation metrics. From the comparison, the consideration of the discriminator was effective for the reduction of both evaluation metrics, especially for FID. The local alignment additionally contributed to the reduction of both per-pixel L1 loss and FID. We also considered the per-pixel L1 loss and FID for grayscale images, blue channel images, and melanin emphasized images by an independent component analysis (ICA)-based method by Tsumura *et al*.^24,25^. The blue channel of color photo images was expected to be closer to UV photo images than grayscale images. In the ICA-based method, a color skin image is decomposed into two types of images by ICA: one indicates melanin component and the other indicates hemoglobin component as shown in Supplementary Fig. 2a,b. By synthesizing the two components with a higher weight for the melanin component, images emphasizing the melanin component are obtained as shown in Supplementary Fig. 2c. We restricted the sum of weights for the melanin and hemoglobin components to two, and selected the weights minimizing the per-pixel L1 loss from a grid search with a step size of 0.1. The selected weights for the melanin and hemoglobin components were 1.3 and 0.7, respectively. In the comparison of these additional cases, blue channel images were closer to the UV photo images than grayscale images in terms of both per-pixel L1 loss and FID. The use of the ICA-based method further reduced both per-pixel L1 loss and FID. Both per-pixel L1 loss and FID for synthetic UV images by UV-photo Net with any condition were lower than those for images by the ICA-based method, blue channel images ,and grayscale images, which supported the effectiveness of UV-photo Net.

Figure 2 shows color photo images (a,d), synthetic UV images generated by UV-photo Net from the color photo images (b,e), and the original UV photo images corresponding to the color photo images (c,f). The subject was in his fifties and agreed to showing his face photo images. The subject was also included in the 24 test samples, and hence his images were not used for training UV-photo Net. Pigment spots identified in the original UV photo images were well reproduced in the synthetic UV images although there exist pigment spots that UV-photo Net failed to reproduce, *e.g.*, the pigment spots in red circles. Of note, pigment areas were also successfully reproduced in the synthetic UV images for profile faces (Fig. 2e) despite the fact that only the color photo images for front faces were used for the training and validation datasets. In addition, UV-photo Net was able to reproduce some of pigment spots that were difficult to be recognized in the color photo images such as those in yellow circles. We also compared synthetic UV images for the subject by UV-photo Net trained without the discriminator or the local alignment (Supplementary Figs. 3 and 4). The comparison confirmed that both the discriminator for CGAN and the local alignment were effective to generate sharp and clear images.

**Figure 3:**
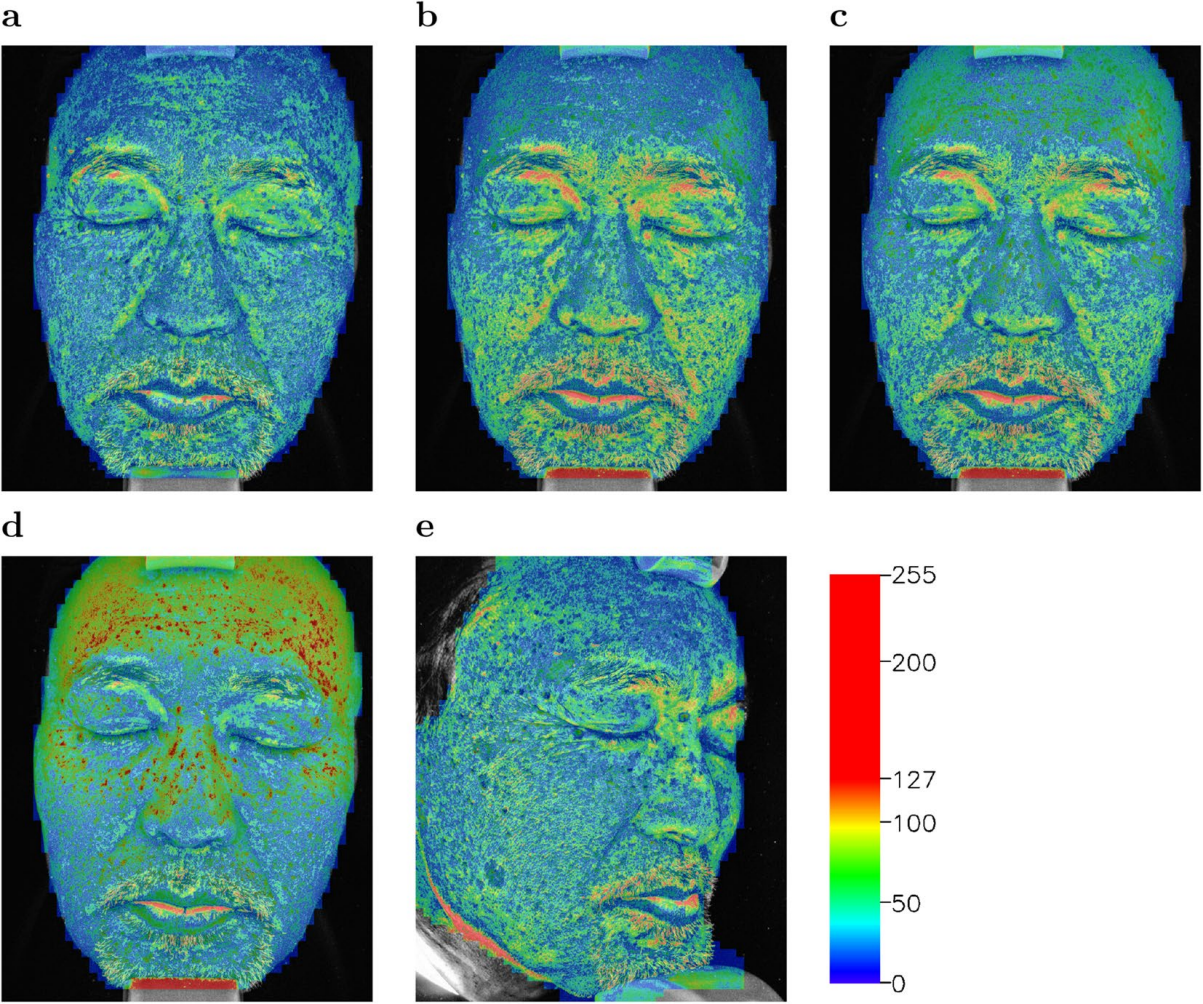
Heatmap images indicating pixel-wise L1 distance from the UV photo image for (**a**) the synthetic UV image by UV-phot Net, (**b**) the image by the ICA-based method, (**c**) the blue channel image, and (**d**) the grayscale image for a front face as well as (**e**) the synthetic UV image by UV-photo Net for a right profile face.

Figure 3 show heatmap images where heatmaps indicating pixel-wise L1 loss from the UV photo image are overlaid on the UV photo image. From the heatmap images for the front face (Fig. 3a–d), we confirmed that the synthetic UV image by UV-photo Net overall has less L1 loss than the images by the ICA-based method, the blue channel image, and the grayscale image. For the heatmap image for the synthetic UV image by UV-photo Net (Fig. 3a), high L1 loss regions were observed around eyes and nasolabial folds, and these regions were blurred in the synthetic UV image in Fig. 2b. Since the specular reflectance in these regions was different from other regions due to 3D facial geometry, UV-photo Net may fail the precise reconstruction of the synthetic image. For the right profile face, shadowed regions caused by the 3D facial geometry such as the region around the right side of the base of the nose and the left part of the face similarly have high L1 losses in the heatmap image (Fig. 3e).

**Table 2:** Per-pixel L1 loss and Fréchet Inception Distance (FID) for synthetic UV images generated by UV-photo Net, grayscale images, blue channel images, and images obtained with the ICA-based method (grayscale and blue channel) ($+$) denotes the case using the corresponding learning technique for training, while (−) denotes the case without the corresponding learning technique.

Method		Discriminator	Local Alignment	Per-pixel L1 Loss	FID
UV-photo Net		(+)	(+)	18.58	50.08
		(−)	(+)	18.92	63.43
		(+)	(−)	18.79	55.84
		(−)	(−)	19.00	66.80
ICA (blue channel)		(−)	(−)	32.08	82.65
ICA (grayscale)		(−)	(−)	48.42	107.29
Blue channel		(−)	(−)	33.38	93.50
Grayscale		(−)	(−)	53.75	120.99

#### Detection of pigment spots in synthetic UV images generated by UV-photo Net

In order to evaluate the practical usefulness of UV-photo Net for pigment spot detection, we performed pigment spot detection for cheek regions in both synthetic UV images by UV-photo Net and UV photo images. For the pigment spot detection, we devised a U-net-based method named Spot Net. In Spot Net, U-net was trained by using UV photo images and their corresponding pigment spot information from the VISIA system.

**Figure 4:**
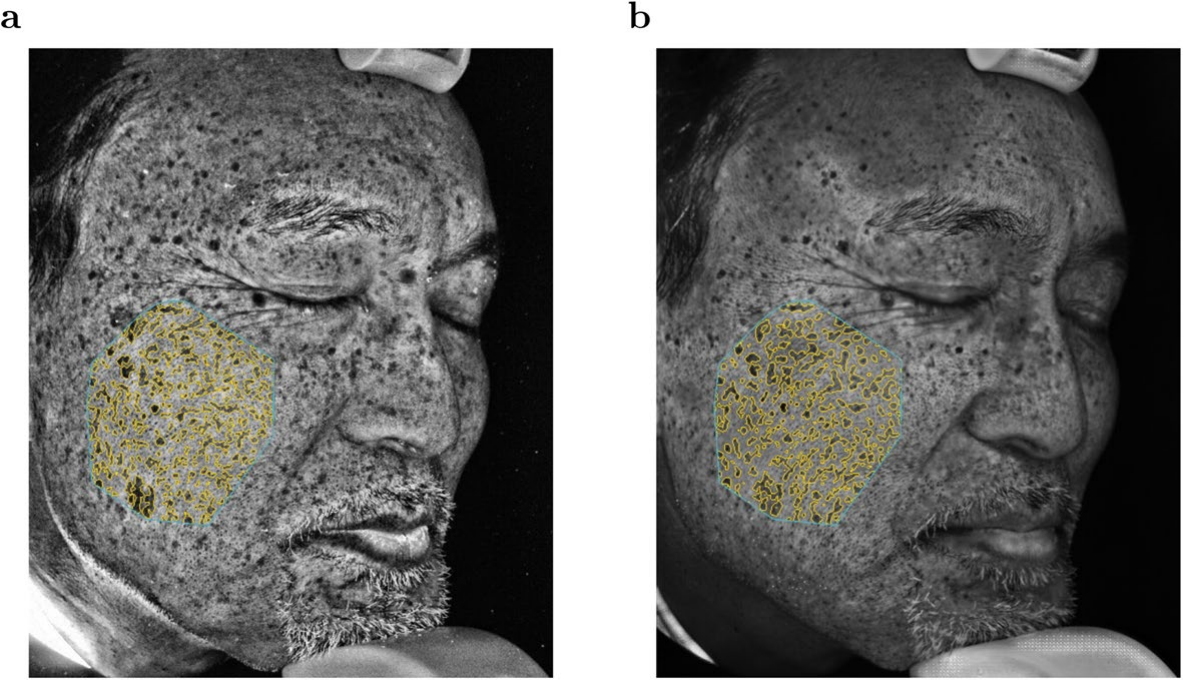
Detected pigment spots by Spot Net for (**a**) the UV photo image of a right profile face and (**b**) the corresponding synthetic UV image by UV-photo Net. The area surrounded by a blue line is a manually selected cheek region, and the areas surrounded by yellow lines denote the detected pigment spots.

We used Spot Net to detect pigment spots in synthetic UV images and UV photo images. Since the VISIA system detects pigment spots in cheek regions only for profile face images, we trained Spot Net using the profile face images for samples included in the training samples. Figure 4a shows pigment spots detected by Spot Net for a cheek region of the UV photo image in Fig. 2f. Spot Net well reproduced pigment spots detected by the VISIA system for the same UV photo image as shown in Supplementary Fig. 5. Figure 4b shows pigment spots detected by Spot Net for the cheek region of the synthetic UV image in Fig. 2e. The pigment spots detected by Spot Net for the synthetic UV image in Fig. 4b formed a similar pattern to those for the UV photo image in Fig. 4a.

**Figure 5:**
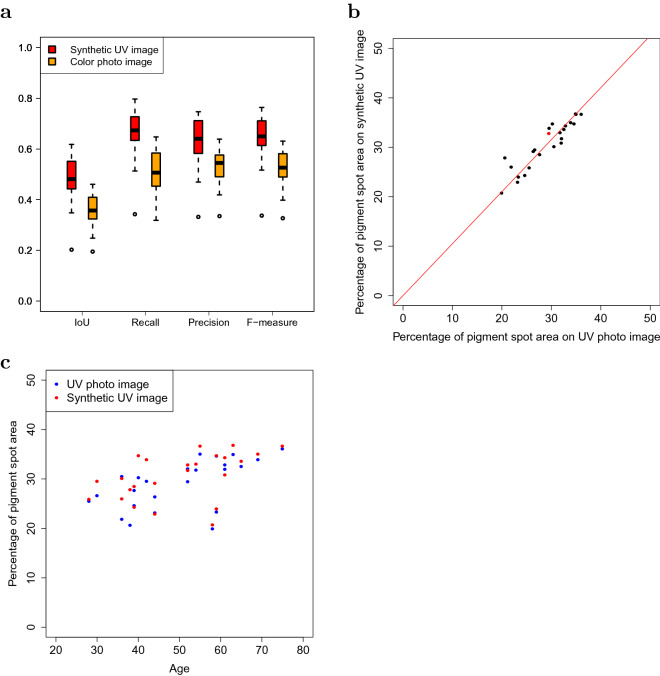
(**a**) Boxplots of IoU, recall, precision, and F-measure of Spot Net for synthetic UV images and color photo images for 24 test samples. (**b**) A plot comparing the percentages of pigment spots in cheek regions between UV photo images and synthetic UV images by UV-photo Net for the test samples. Dots indicate pairs of the percentages, and the red line is a linear regression line without intercept for the pairs of the percentages. The regression coefficient is 1.05. The red dot indicates the pair of the percentages for the subject in Fig. 4. (**c**) A plot showing the relationship between the age and percentage of pigment spot areas for UV photo images and synthetic UV images by UV-photo Net for the test samples. The x-axis indicates the age of the samples, and the y-axis indicates the percentage of pigment spot areas in the cheek regions for the UV photo images and synthetic UV images of the respective samples.

We used Spot Net to detect pigment spots in the cheek regions of front face UV photo images and their corresponding synthetic UV images generated by UV-photo Net for the 24 test samples. In order to consider the pigment spot detection directly from color photo images, we also prepared Spot Net that was trained by using color photo images as input images instead of UV photo images. Figure 5a shows boxplots for intersection over union score (IoU), recall, precision, and F-measure obtained by designating pigment spots detected from the UV photo images as true pigment spots. IoU is a metric used for the evaluation on object detection and obtained by calculating the size of the intersection of true and estimated regions divided by the size of their union. F-measure is given by the harmonic mean of recall and precision and used to evaluate the aggregated accuracy considering both recall and precision. For IoU, recall, precision, and F-measure, the maximum and minimum values are 1 and 0, and the higher value is better. The results of synthetic UV images were better than those for color photo images for all IoU, recall, precision, and F-measure. Although the synthetic UV images were also obtained from the color photo images, the information from the UV-photo images via UV-photo Net presumably contributed to the more accurate pigment spot detection.

We measured the percentages of pigment spot areas detected by Spot Net in the cheek regions of the UV photo images and synthetic UV images for the 24 test samples (Fig. 5b). The percentages for the UV photo images and synthetic UV images were highly correlated (Pearson’s correlation coefficient 0.92, p-value = $3.43 \times 10^{-10}$). The red line in Fig. 5b indicates the linear regression without intercept for the pairs of the percentages, for which the regression coefficient is 1.05. Since the percentage of the pigment spot areas was highly correlated between UV photo images and synthetic UV images, the size of pigment spot areas in the UV photo images can be estimated by evaluating synthetic UV images. Also, the tendency in the increase of the percentage of pigment spot areas along with the age was observed for both UV photo images and synthetic UV images (Fig. 5c).

#### Verification of synthetic UV images for smartphone photo images

**Figure 6:**
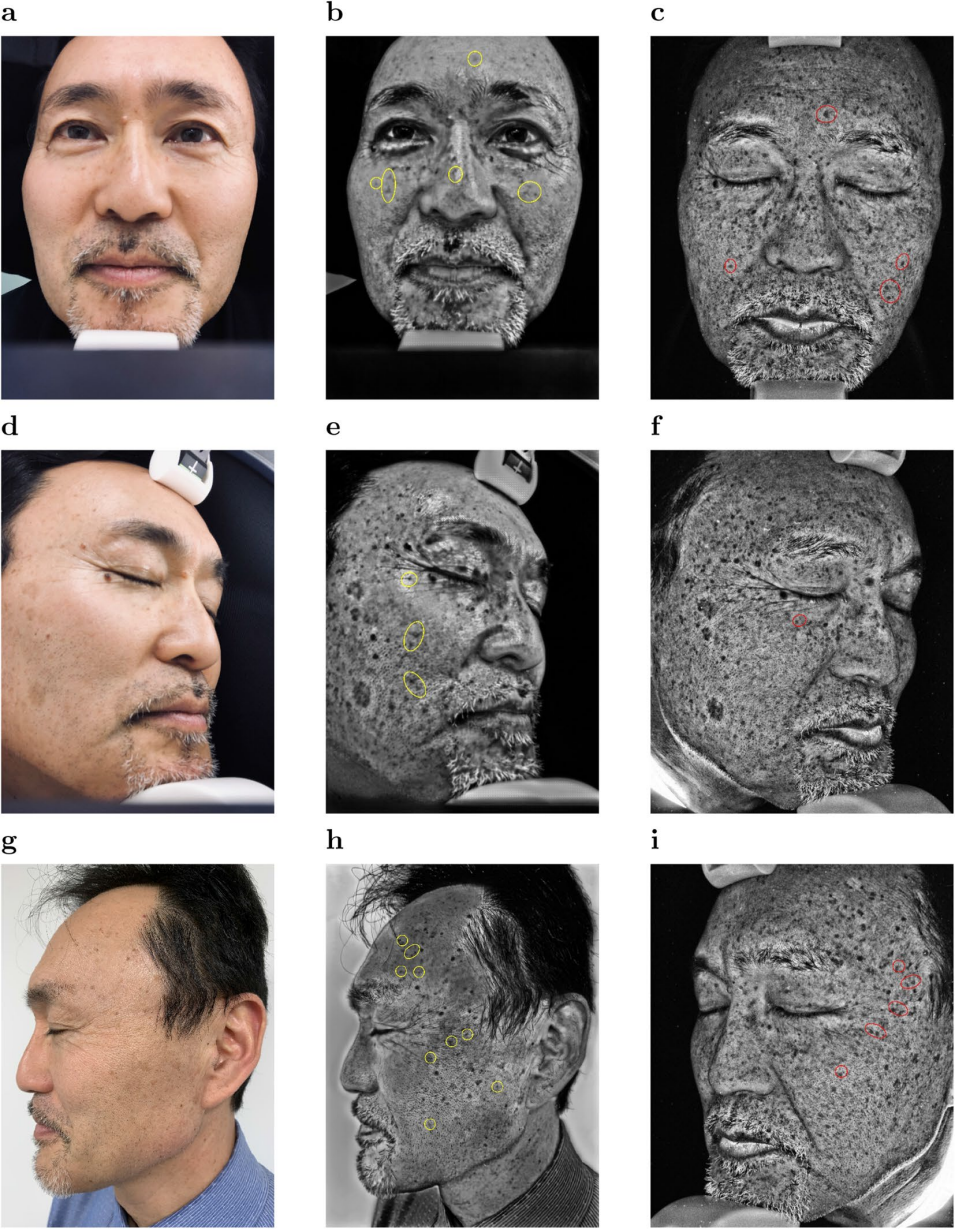
Color photo images for front, right profile, and left profile faces taken by iPhone (**a**,**d**,**g**), their respective synthetic UV images by UV-photo Net (**b**,**e**,**h**) from the iPhone photo images, and UV photo images for the front, right profile, and left profile faces taken by the VISIA system (**c**,**f**,**i**).

We applied UV-photo Net to front, right profile, and left profile face photo images taken by iPhone (Fig. 6a,d,g). The width and height of iPhone images was $2320\times 3088$ or $3024\times 4032$. The photo images for the front face and the right profile face were taken by putting iPhone near the camera position of the VISIA system. In order to examine the influence of lightning conditions to UV-photo Net, the iPhone image for the left profile face was taken under a standard fluorescent light. Figure 6b,e,h show synthetic UV images by UV-photo Net from the iPhone images in Fig. 6a,d,g, respectively. Two board-certified dermatologists evaluated the synthetic UV images for the iPhone images through the comparison with the original iPhone images (Fig. 6a,d,g) and the UV photo images taken with the VISIA system (Fig. 6c,f,i). Red circles indicate examples of pigment spots that UV-photo Net failed to reproduce, and yellow circles indicate examples of pigment spots that were difficult to be recognized in the iPhone images by the two dermatologists.

The iPhone image for the front face is not well-focused, and hence pigment spots were not reproduced clearly in the corresponding synthetic UV image. On the other hand, the iPhone image for the right profile face is clear, and the pigment spots were well reproduced in the synthetic UV image for the right profile face. The synthetic UV image for the left profile face is less realistic somewhat as the UV photo image due to the difference of the lightning condition. However, the pigment spots were well reproduced in the synthetic UV image because of the clear photographing of the left profile face in the iPhone image. While we demonstrated that UV-photo Net was also effective for the pigment spot detection in the iPhone images, we confirmed the importance of stable environments for taking well-focused photo images for the accurate pigment spot detection from smartphone images.

## Conclusion

We proposed a CGAN-based deep learning method named UV-photo Net to estimate UV photo images from color photo images for the daily care of UV skin damages. We evaluated UV-photo Net using color and UV photo face images of 150 individuals for a training dataset, those of 10 individuals for a validation dataset, and those of 24 individuals for a test dataset. Although the number of individuals considered in the training set seems insufficient for training deep learning models, the deep learning models comprising UV-photo Net were trained using not whole images but image patches, and the sufficient number of image patches were obtained for training the models. As a result, UV-photo Net in our experiment was able to generate synthetic UV images that were much closer to UV-photo images than simple grayscale images, blue channel images, and images obtained with the ICA-based method in terms of both per-pixel L1 loss and FID. In order to fix the mismatch between color and UV photo images in the training data, we devised a local alignment method. We confirmed that the consideration of both CGAN and the local alignment was effective to improve the quality of synthetic UV images.

In order to examine the effectiveness of UV-photo Net for pigment spot detection, we proposed a deep learning-based pigment spot detection method named Spot Net. We used Spot Net to analyze UV skin damages for UV photo images and synthetic UV photo images by UV-photo Net in terms of the percentages of detected pigment spot areas in cheek regions. These percentages were highly correlated (Pearson’s correlation coefficient 0.92, p-value = $3.43 \times 10^{-10}$), and hence the percentage for the synthetic UV image can be used for as an indicator of skin damage with sufficient accuracy. We also applied UV-photo Net to iPhone images in order to examine the practical usefulness of UV-photo Net in smartphone camera images. The validation by two dermatologists showed that the resulting synthetic UV images successfully emphasized pigment spots which were difficult to be recognized in the original iPhone images.

In contrast to the above successful points, UV-photo Net still has some limitations. The estimated results for shadowed regions such as regions around eyes or nasolabial holds, were relatively less accurate than other regions due to the difference of skin specular reflectance. Furthermore, the resulting synthetic UV image by UV-photo Net was less realistic for iPhone image taken under the different lightning condition from the training dataset. To address the influence of skin specular reflectance, Gevaux *et al*. proposed to the additional use of artificial training data by simulating the effect of varying lighting and viewing conditions as a data augmentation process^26^. We believe that both issues can be resolved by additionally using the training data under various conditions similarly to their solution. Although pairs of color and UV photo images are required in the learning framework considered in this study, color photo images without the corresponding UV photo images can be used for training under CycleGAN framework^27^. The hybrid use of such a learning framework is promising for the increase of the training data under various conditions, and will be a possible future work for more robust estimation.

There exist a number of studies analyzing genetic aspects of sunburn related diseases. Genome-wide association studies, for example, revealed the genes related to melanin production and sunburn, and those genes are also related to actinic keratosis, squamous cell carcinoma, basal cell carcinoma, and melanoma^28^. Sun protection and care for UV skin damages in early stages are essential for people genetically susceptible to sunburn related diseases. Since the understanding of skin damage from UV photo image promotes the use of sunscreen^3^, further development of our method with the consideration of additional deep learning technologies such as CycleGAN is important for enabling the daily basis care of UV skin damages and skin cancers especially for the people genetically susceptible to sunburn.

## Methods

### Study participants and ethical committee approval

This study was approved by the ethical committee of the Tohoku University Graduate School of Medicine. The participants of this study is comprised of 184 Japanese individuals (180 females and 4 males) who visited the Dermatology Clinic of the Tohoku University Hospital (Sendai, Japan) from 2013 to 2019. Written informed consent was obtained from all the study participants to use their color and UV photo images. Color and UV photo image pairs for the 184 individuals were taken with VISIA Skin Analysis System (Canfield Imaging Systems, Fairfield, NJ, United States)^21^ in the Dermatology Clinic of Tohoku University hospital through the general clinical practice. We used standard mode for photographing color images. The room for photographing was maintained with a constant temperature at 26 degrees Celsius and 31% humidity without daylight inside^29^. All methods and procedures follow the Declaration of Helsinki and were carried out in accordance with the Japanese guidelines and regulations for medical researches. We divided the 184 individuals into 160 individuals as training samples and the remaining 24 individuals as test samples. A male study participant in the test samples agreed to the publication of his photo images in online open-access publications.

### Detection of face region to designate training and evaluation area (Fig. 1**a**-1)

We trained a deep neural network model called Inception-v4^30^ for binary classification to detect face regions, from which we extracted image patches of $256\times 256$ pixels for training UV-photo Net. Since image patches of $256\times 256$ pixels used in UV-photo Net are too coarse to detect face regions precisely in our image dataset, we used smaller image patches of $120\times 120$ pixels for input images of Inception-v4. For the generation of image patches of $120\times 120$ pixels for training, we first detected face landmarks for eyes, eye brows, nose, lips, and jawline from color photo images as shown in Supplementary Fig. 1a by using a method based on an ensemble of regression trees^31^ implemented in dlib library (http://dlib.net/). We then extracted image patches of $120 \times 120$ pixels labeled as face region from the region surrounded by the detected face landmarks. We also extracted image patches of $120 \times 120$ pixels for non-face region from the outside region of the jawline estimated by the face landmark detection (the hatched region in Supplementary Fig. 1a). Although the detection of some of the face regions such as forehead regions is difficult by the face landmarks, the Inception-v4-based method is expected to detect all the face regions accurately. We used around 30,000 image patches of $120 \times 120$ pixels and their associated labels for training Inception-v4. Overlapping between the image patches was allowed in the extraction. In the training process, parameters of Inception-v4 were updated iteratively with 100,000 steps by using Adam solver^32^ with a learning rate of 0.0001 and a batch size of 100. After the training, we generated face region maps for all the color photo images as shown in Supplementary Fig. 1b by assembling image patches of $120\times 120$ pixels with the estimated labels by the trained Inception-v4 model.

### Structure and training of UV-photo Net

**Figure 7:**
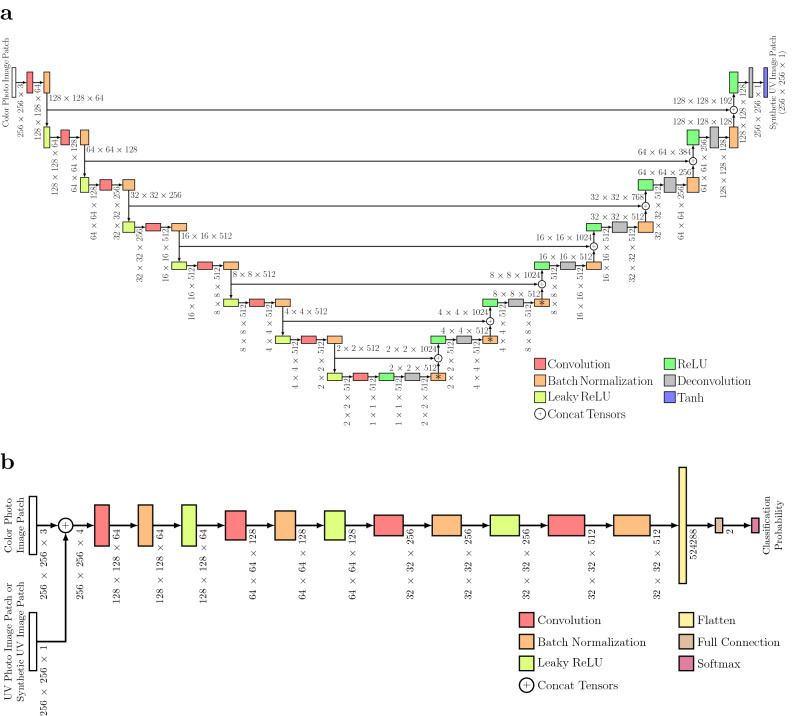
Structures of deep neural network models for the generator and discriminator. (**a**) The structure of U-net used for the generator in UV-photo Net. (**b**) The structure of the model for the discriminator in UV-photo Net. The value beside the outgoing arrow of each layer denotes the shape of the output tensor. The number of channels and image size of tree-dimensional output tensors are represented by the width and height of their rectangles, respectively. For vector-shaped output tensors, the height of their rectangles represents their respective vector length. The width of rectangles for the vector-shaped output tensors is equivalent to the size for one channel. Dropout was applied to the output tensors for the rectangles with ‘*’.

We developed UV-photo Net, which converts color photo images to synthetic UV photo images using a deep neural network model trained under CGAN. CGAN considers two types of models called generator and discriminator. We used a deep neural network model called U-net for the generator. The structure of U-net used in UV-photo Net is shown in Fig. 7a, where the height *h*, the width *c*, and the number of channels *c* of the output tensor of each layer are denoted by $h \times w \times c$ beside the outgoing arrow. The shape of the output tensor is also visually represented by the rectangle size of the layer in a similar manner to the original article describing the U-net structure^20^. Dropout^33^ with a dropout rate of 0.5 was applied to output tensors for the rectangles with ‘$*$’ in the training process. In U-net, input images are encoded to smaller images in the first half part of the network structure. Since global characteristics of the input images can be captured by encoded images, U-net can generate images considering both local and global characteristics by additionally using encoded images in the latter half part of the network structure. Although whole color photo images are too large to handle directly in U-net, we used U-net to convert color photo image patches of $256\times 256$ pixels into synthetic UV image patches of $256\times 256$ pixels. A whole synthetic UV image was then reconstructed by assembling the synthetic UV image patches.

For the discriminator, we used a deep neural network that takes two types of input images; one is a color photo image patch, and the other is its corresponding UV photo image patch or a synthetic UV image patch generated from the color photo image patch by the generator (Fig. 7b). The discriminator is trained to classify whether the latter input images are UV photo image patches or synthetic UV image patches from the generator. The generator is trained based on the per-pixel L1 loss between synthetic UV image patches and the original UV photo image patches as well as the loss based on the classification results from the discriminator for the synthetic UV image patches. By alternately training the generator and the discriminator, the generator comes to generate synthetic UV image patches that are difficult to be distinguished by the discriminator, while the discriminator comes to successfully classify the synthetic UV image patches that are perceptually realistic as UV photo image patches.

#### Formal description for training process

We let *G*(*X*) be a function representing the generator that generates a synthetic UV image patch from color photo image patch *X*. We also let *D*(*X*, *Y*) be a function representing the discriminator that returns the probability of *Y* being the UV photo image patch corresponding to color photo image patch *X*. The ideal *D*(*X*, *Z*) should return one if *Y* is the UV photo image patch corresponding to *X* and zero otherwise. Let *I* and *J* be sets of indices for training images. The following equation is optimized in training under CGAN:

$$\begin{aligned} L_{G,D} = \min _{G} \max _{D} \frac{1}{|I|} \sum _{i \in I} \left( \frac{1}{w_i \cdot h_i} |Y_i - G(X_i)| + \lambda \log (1 - D(X_i, G(X_i))\right) + \frac{\lambda }{|J|} \sum _{j \in J} \log D(X_j, Y_j), \end{aligned}$$

where $Y_i$ is the UV photo image patch that corresponds to color photo image patch $X_i$, $w_i$ and $h_i$ respectively are the width and height of $Y_i$, $\lambda$ is a weight for the loss from the discriminator, and $|\cdot |$ denotes the size of the set. We empirically chose 0.005 as the value of $\lambda$ for UV-photo Net. The optimization is performed by generator *G* and discriminator *D*, alternately. For the optimization by the discriminator, the following cost function is minimized with respect to *D*:

$$\begin{aligned} - \frac{1}{|I|} \sum _{i \in I} \log (1 - D(X_i, G(X_i))) - \frac{1}{|J|} \sum _{j \in J} \log D(X_j, Y_j). \end{aligned}$$

For the optimization by the generator, the following cost function is minimized with respect to *G*:

$$\begin{aligned} \sum _{i \in I} \left( \frac{1}{w_i \cdot h_i} |Y_i - G(X_i)| + \lambda \log (1 - D(X_i, G(X_i))\right) , \end{aligned}$$

where the first term is the per-pixel L1 loss of UV photo image patch $Y_i$ and synthetic UV image patch $G(X_i)$, and the second term is the loss for the naturalness of synthetic UV image patch $G(X_i)$ as a UV photo image patch evaluated by discriminator *D*. Under the alternate update of the generator and the discriminator, the generator can generate synthetic UV images that are close to the original UV photo images in terms of per-pixel L1 loss and perceptually realistic as UV photo images. It is worth noting that sets of indices *I* and *J* should be disjoint for stable and proper training because the discriminator can easily distinguish synthetic UV image patches from UV photo image patches in the cost function for the generator if indices in *I* are also included in *J*.

#### Local alignment of color and UV photo images for training data (Fig. 1**a**-2-ii)

Color photo images and their corresponding UV photo images are not completely matched in pixel-level due to the subtle movement of subjects in photographing time interval. Such pixel-level mismatches can cause vague and blurred areas in synthetic UV images. We thus considered the alignment of color and UV photo images to correct the pixel-level mismatches. The alignment with the whole image was not suitable for the UV-photo Net since the pixel-level mismatches occur locally. It is also difficult to directly align the color and UV photo images due to the high discrepancy in their characteristics of images and pixel-level information. A possible solution for the former issue is to align image patches instead of whole images. For the latter issue, we instead aligned UV photo images and synthetic UV images for the corresponding color photo images obtained by UV-photo Net trained without the discriminator and the local alignment. Since image patches of $256\times 256$ pixels are too small to capture the outlines of facial parts such as eyebrows, eyes, nose, and lips, we used larger image patches of $900\times 900$ pixels for the alignment. Image patches of $900\times 900$ pixels were extracted in a tiled manner where overlapping of the image patches was allowed. We considered vertical and horizontal shifts in the alignment and selected the shift with the minimum mean per-pixel L1 loss between UV photo and synthetic UV image patches of $900\times 900$ pixels as the alignment result. The alignment results for synthetic UV image patches of $900\times 900$ pixels were directly applied to the corresponding color photo image patches of $900\times 900$. We then aligned each image patch of $256\times 256$ pixels for training U-net using the alignment result of the image patch of $900\times 900$ pixels with the minimum distance with the image patch of $256\times 256$ pixels. We defined the distance between an image patch of $900\times 900$ pixels and an image patch of $256\times 256$ pixels by the distance of their center points.

#### Training process of UV-photo Net (Fig. 1**a**-2-i,iii)

We extracted small image patches of $256\times 256$ pixels from the face regions of the training and validation datasets. The face regions were detected by the aforementioned Inception-v4-based method. We considered random vertical flip, horizontal flip, and 90-degree rotation as the data augmentation. For training under CGAN, models for the generator and discriminator were alternately updated with 150,000 iterations. We considered early stopping to select the best model according to the mean pixel-wise L1 loss in the validation dataset. We then updated only the model for the discriminator with 5,000 iterations, and selected the best model for the discriminator according to the discriminator loss in the validation dataset. We further updated only the model for the generator with 5,0000 iterations and selected the best model for the generator according to the mean pixel-wise L1 loss in the validation dataset. For all the iterations, we set batch size to 80 and used Adam solver with a learning rate of 0.0001 to update parameters. In order to examine the effect of the discriminator for CGAN, we also considered UV-photo Net trained without the discriminator for which only the model for the generator was trained under the loss function with $\lambda = 0$. In the UV-photo Net trained without the discriminator, we only updated the model for the generator with 150,000 iterations. Early stopping based on the mean per-pixel L1 loss for the validation set was also considered for the UV-photo Net trained without the discriminator.

### Pigment spot detection by Spot Net

We devised a U-net-based method for pigment spot detection named Spot Net. The structure of U-net for Spot Net is the same as the structure of U-net for UV-photo Net except for the final output layer. The softmax function for binary classification was instead used in the final output layer for Spot Net. For training Spot Net, the combination of the cross entropy of the softmax function and the DICE coefficient^34^ was used as the cost function. Since the VISIA system detects pigment spots in cheek regions only for profile face images, we trained Spot Net using UV photo image patches of $256\times 256$ pixels from the cheek regions of the profile face images and their corresponding spot map image patches. We also included synthetic UV image patches by UV-photo Net as the input images of the training dataset to adapt Spot Net for the synthetic UV images as well. Of the 160 training samples, 69 samples had profile face images with pigment spot information detected by the VISIA system. We used profile face images for 49 samples as a training dataset and profile face images for the remaining 20 samples as a validation dataset for Spot Net. We set batch size to 80 and used Adam solver with a learning rate of 0.0001 to update parameters with 150,000 iterations. The model with the minimum cost in the validation dataset was used as the training result. For the data augmentation, we considered random brightness change as well as random vertical flip, horizontal flip, and 90-degree rotation. In order to consider the pigment spot detection directly from color photo images, we also prepared Spot Net that was trained by using color photo image patches as the input images of the training and validation datasets.

### Implementation and computational resources for proposed methods

We implemented the deep neural network models for face region detection, UV-photo Net, and Spot Net in Python 3 and TensorFlow r.1.15. We used GeForce GTX 1080 and GeForce RTX 2080 SUPER for GPU computation to train and test these deep neural network models.

## Supplementary Information

Supplementary Information 1.
