# Supplementary material for: Facial UV photo imaging for skin pigmentation assessment using conditional generative adversarial networks
Source: Sci Rep. 2021 Jan 13;11:1213. doi: 10.1038/s41598-020-79995-4 (PMC7806902; doi:10.1038/s41598-020-79995-4)
Supplement: Supplementary file 1 — Supplementary Information 1. [file 41598_2020_79995_MOESM1_ESM.pdf]

# Supplementary material for “Facial UV photo imaging for skin pigmentation assessment using conditional generative adversarial networks”

Kaname Kojima[1,2], Kosuke Shido[3], Gen Tamiya[1,2], Kenshi Yamasaki[3], Kengo Kinoshita[1,4,5,6], Setsuya Aiba[3]

**1** Tohoku Medical Megabank Organization, Tohoku University, Sendai, Miyagi Japan

**2** RIKEN Center for Advanced Intelligence Project, Chuo-ku, Tokyo Japan

**3** Department of Dermatology, Tohoku University Graduate School of Medicine, Sendai, Miyagi Japan

**4** Tohoku University Graduate School of Information Sciences, Sendai, Miyagi Japan

**5** Institute of Development, Aging and Cancer, Tohoku University, Sendai, Miyagi Japan

**6** Advanced Research Center for Innovations in Next-Generation Medicine, Tohoku University, Sendai, Miyagi Japan

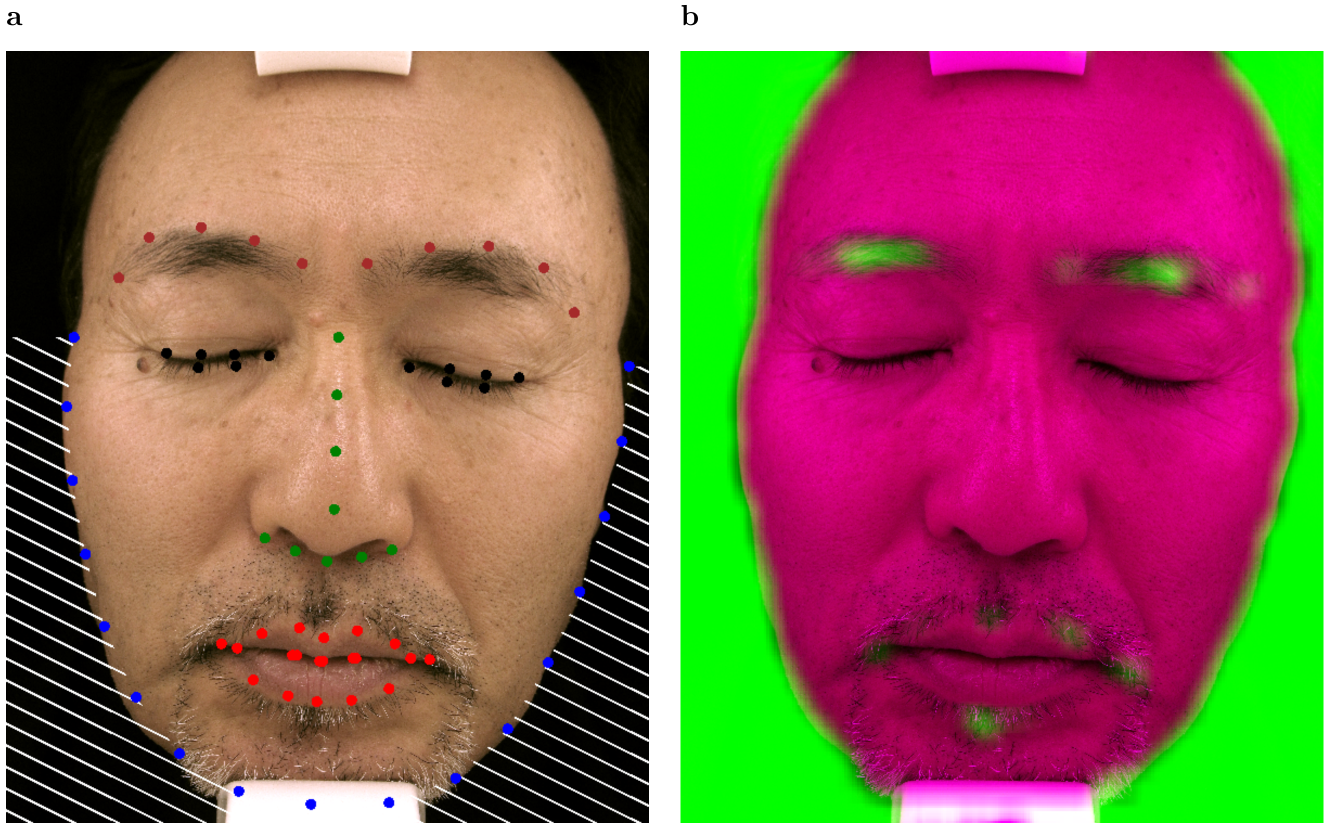

Supplementary Figure 1: (a) Face landmarks such as eyes, eyebrows, nose, lips, and jawline detected by a method based on an ensemble of regression trees implemented in dlib library (http://dlib.net/). The hatched region represents the outside region of the jawline, from which non-face small image patches were extracted for the training data of face region detection. (b) The region highlighted in red represents the face region detected by Inception-v4.

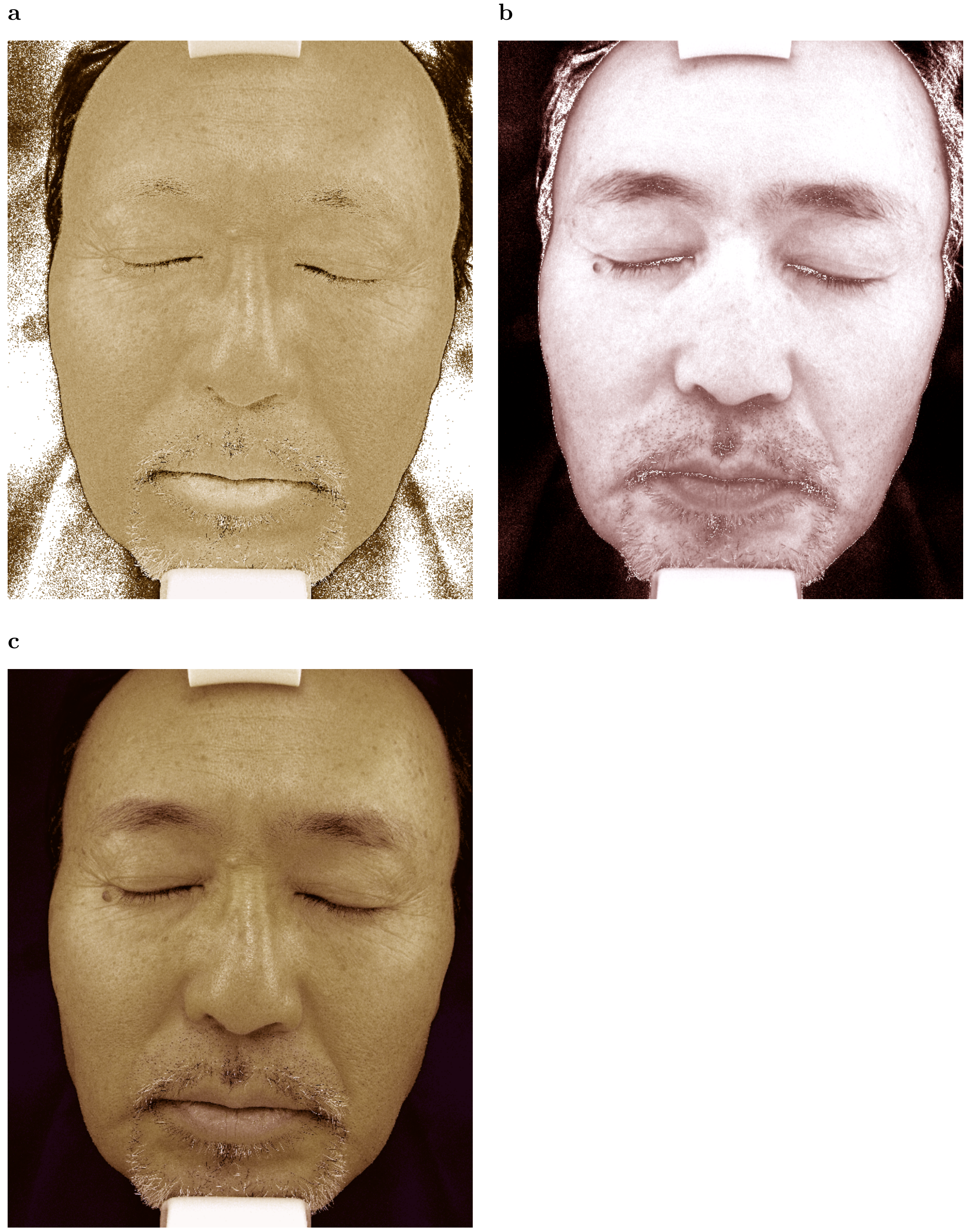


**Supplementary Figure 2**: (**a**) Melanin component and (**b**) hemoglobin component images of a color photo face image obtained by an independent component analysis (ICA)-based image decomposition method in [1, 2]. (**c**) The image obtained by the synthesis of these two components. In order to emphasize the melanin component, a higher weight was given to the melanin component than the hemoglobin component in the synthesis.

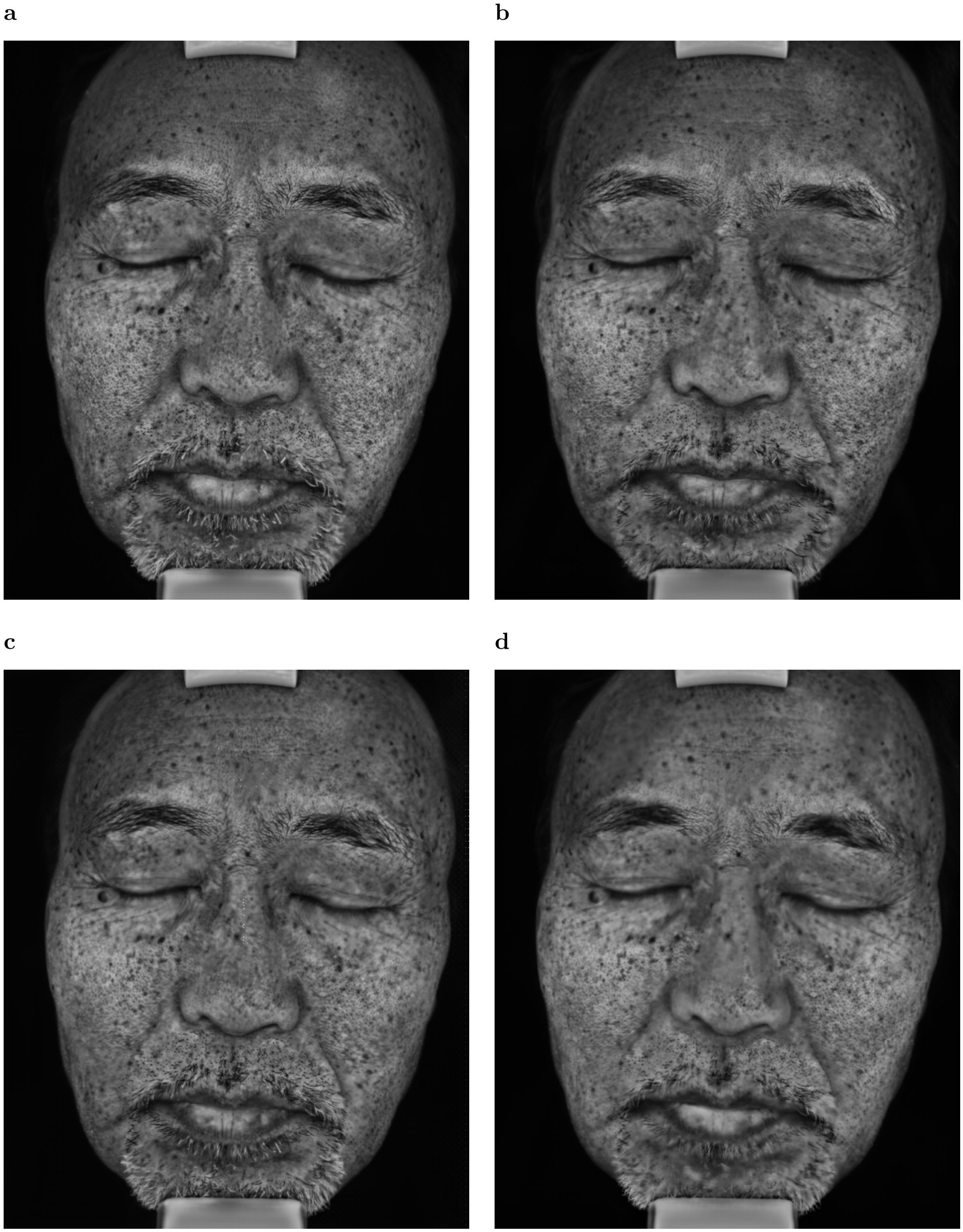


**Supplementary Figure 3**: (**a**) A synthetic UV front face image generated by UV-photo Net. (**b**) A synthetic UV front face image generated by UV-photo Net without the discriminator. (**c**) A synthetic UV front face image generated by UV-photo Net without the local alignment. (**d**) A synthetic UV front face image generated by UV-photo Net without the discriminator and local alignment. CGAN is effective for clarifying synthetic UV images visually, especially the area around nose from the comparison of the synthetic UV images. Local alignment is also effective for clarifying synthetic UV images visually.

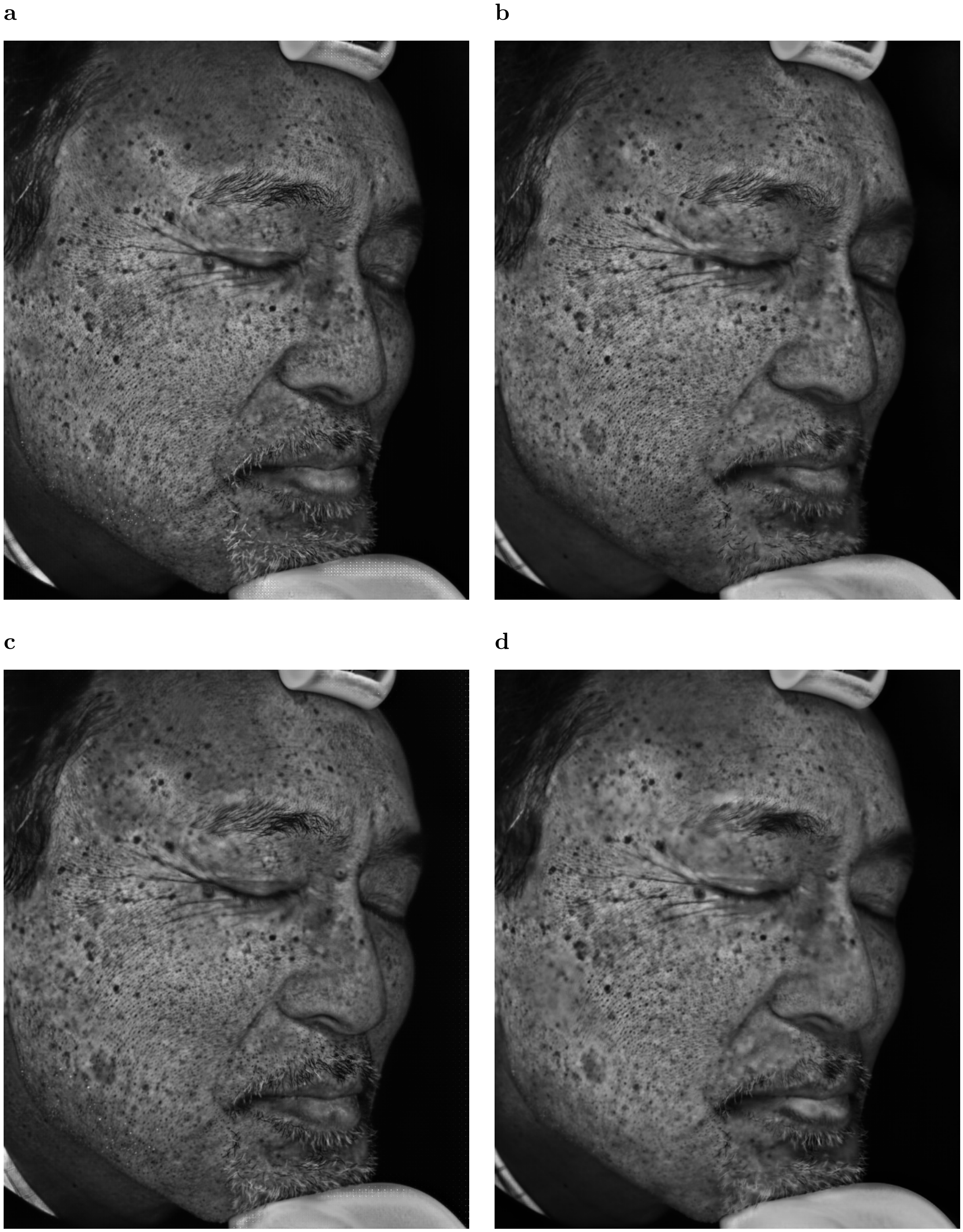


**Supplementary Figure 4**: (**a**) A synthetic UV profile face image generated by UV-photo Net. (**b**) A synthetic UV profile face image generated by UV-photo Net without the discriminator. (**c**) A synthetic UV profile face image generated by UV-photo Net without the local alignment. (**d**) A synthetic UV profile face image generated by UV-photo Net without the discriminator and local alignment. CGAN is effective for clarifying synthetic UV images visually, especially from the comparison of the synthetic UV images. Local alignment is also effective for clarifying synthetic UV images visually.

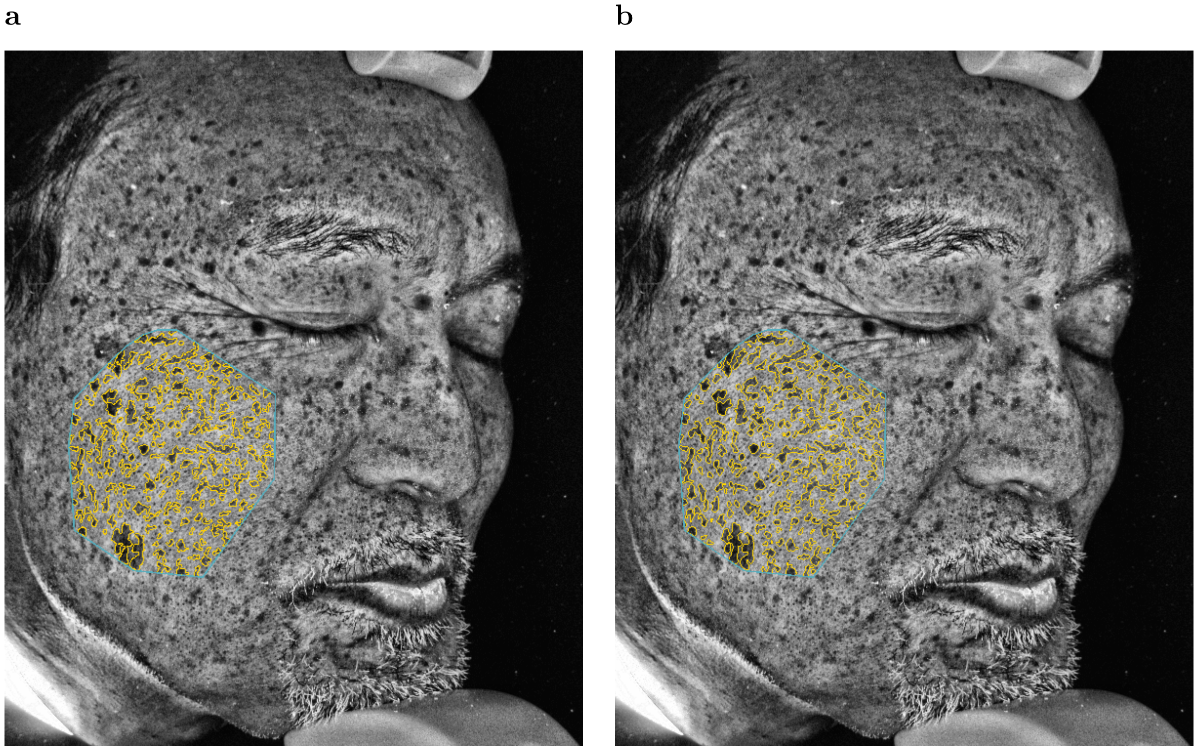


**Supplementary Figure 5**: (**a**) Pigment spots detected by the VISIA system for a cheek region of a UV photo image for a profile face. (**b**) Pigment spots detected by Spot Net for the cheek region of the same UV photo image.

**Supplementary Table 1**: Per-pixel L1 losses calculated by the mean of pixel-wise absolute distance for synthetic UV images generated by UV-photo Net, grayscale images, blue channel images, and images obtained with the ICA-based method (grayscale and blue channel). (+) denotes the case using the corresponding learning technique for training, while (−) denotes the case without the corresponding learning technique.

| Method | Discriminator | Local Alignment | Per-pixel L1 Loss |
|---|---|---|---|
| UV-photo Net | (+) | (+) | 22.90 |
| | (−) | (+) | 23.02 |
| | (+) | (−) | 23.07 |
| | (−) | (−) | 23.15 |
| ICA (blue channel) | (−) | (−) | 38.36 |
| ICA (grayscale) | (−) | (−) | 52.92 |
| Blue channel | (−) | (−) | 39.50 |
| Grayscale | (−) | (−) | 58.23 |

# References

[1] Tsumura, N., Haneishi, H., and Miyake, Y., Independent-component analysis of skin color image, *Journal of the Optical Society of America A*, 16(9): 2169–2176, 1999.

[2] Tsumura, N., Haneishi, H., and Miyake, Y., Independent component analysis of spectral absorbance image in human skin, *Optical Review*, 7(6): 479–482, 2000.
